# 2D array of cold-electron nanobolometers with double polarised cross-dipole antennas

**DOI:** 10.1186/1556-276X-7-224

**Published:** 2012-04-18

**Authors:** Leonid S Kuzmin

**Affiliations:** 1 Chalmers University of Technology, Gothenburg, S-41296, Sweden

**Keywords:** Cold-electron bolometer, 2D array, Focal plane antenna, SIN tunnel junction, Andreev contact, JFET readout, 85.25.Oj, 85.25.Pb, 74.50. + r

## Abstract

A novel concept of the two-dimensional (2D) array of cold-electron nanobolometers (CEB) with double polarised cross-dipole antennas is proposed for ultrasensitive multimode measurements. This concept provides a unique opportunity to simultaneously measure both components of an RF signal and to avoid complicated combinations of two schemes for each polarisation. The optimal concept of the CEB includes a superconductor-insulator-normal tunnel junction and an SN Andreev contact, which provides better performance. This concept allows for better matching with the junction gate field-effect transistor (JFET) readout, suppresses charging noise related to the Coulomb blockade due to the small area of tunnel junctions and decreases the volume of a normal absorber for further improvement of the noise performance. The reliability of a 2D array is considerably increased due to the parallel and series connections of many CEBs.

Estimations of the CEB noise with JFET readout give an opportunity to realise a noise equivalent power (NEP) that is less than photon noise, specifically, NEP = 4 10^−19^ W/Hz^1/2^ at 7 THz for an optical power load of 0.02 fW.

## Background

Cosmic microwave background (CMB) measurements were ranked second by *the journal Science* among the top 10 Achievements of the Decade [1]. In 2000 and 2003, an experiment known as Balloon Observations of Millimetric Extragalactic Radiation and Geophysics (BOOMERanG) measured the CMB in detail in patches of the sky [2]. Then in 2003, NASA's space-based Wilkinson Microwave Anisotropy Probe mapped the CMB across the sky, producing an exquisite “baby” picture of the cosmos. These and the measurements that followed have started to transform cosmology from a largely qualitative endeavour to a precision science with a standard theory, known as Precision Cosmology [1,2]. Recent cosmology experiments have discovered that the universe mainly consists of dark energy and dark matter. Several cosmology instruments (e.g. BOOMERanG-3, SPICA (Space Infra-Red Telescope for Cosmology and Astrophysics telescope) [3], MILLIMETRON [4], and B-POL) are currently being designed to resolve the nature of these dark components. The science of SPICA and MILLIMETRON will include the cool universe, encompassing galaxy formation in the early distant universe, star formation in the local universe and planet formation in our own galaxy. The observing capabilities on the aforementioned instruments will extend to wavelengths shorter than those on Herschel.

The experiments will require a new generation of ultra-sensitive detectors with exceptionally low noise equivalent power (NEP) in the range of 10^−19^–10^−20^ W Hz^−1/2^[3]. Superconducting detectors with nanoscale absorbers are the most promising candidates for the next generation of supersensitive bolometers. At present, the most widespread superconducting bolometer is a transition-edge sensor (TES) [5,6]. However, due to artificial DC bias heating, the TES has increased temperature of operation, excess noise and strictly limited saturation power.

The novel concept of the cold-electron bolometers (CEB) [7,8] has been invented to overcome these problems. The CEB concept is based on a unique combination of the RF (radio frequency) capacitive coupling of the absorber to the antenna through the capacitance of the superconductor-insulator-normal (SIN) tunnel junctions [7] and the direct electron cooling of the absorber by the same SIN tunnel junction [8]. The noise properties of this device are improved considerably by decreasing the electron temperature. Direct electron cooling leads to a considerable increase in the saturation power due to the removal of the incoming power from the sensitive nanoabsorber. The direct electron cooling also provides strong negative electrothermal feedback for the signal [9], analogous to the TES [5]; but here, the artificial DC heating is replaced by direct electron cooling from the SIN tunnel junctions [10-12] to a minimum temperature (possibly less than phonon temperature). This concept can lead to a major breakthrough in the realisation of supersensitive detectors.

The CEB can be used with both SQUID readout [7-9,13-15] and JFET/CMOS (Complementary metal–oxide–semiconductor) [9,14-16]. The JFET and CMOS readouts (with multiplexing) have been used in Herschel and BOOMERanG with high-ohmic Ge boometers. The SQUID readout (with multiplexing) is in a stage of development with low-ohmic TES. The overall goal is to achieve, with a CEB readout from JFET or SQUID (Superconducting quantum interference device), an NEP that is less than the photon noise with a low optical power load for SAFARI (SpicA FAR-infrared instrument) and MILLIMETRON.

### 2D array of the CEB with a double polarisation cross-dipole antennae

**Figure 1:**
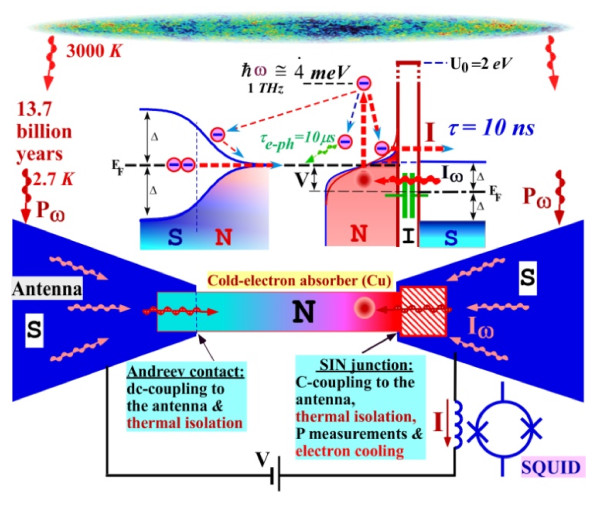
**Schematic of the optimal CEB with capacitive coupling to the antenna [13].** A normal nanoabsorber is coupled to the antenna through the capacitance of an SIN tunnel junction and through an SN Andreev contact. The SIN tunnel junction is also used for thermal isolation, electron cooling and for reading the signal with a SQUID or a JFET. The optical power load is relatively low: *P*_0_ = 0.02 fW. The photon noise is $NEP_{\text{phot}}=\sqrt{2P_{0}*hf}$_**.**_ For the 7 THz channel, $NEP_{\text{phot}}=4\times 10^{-19}{\text{WHz}}^{-1/2}$.

The CEB is a planar antenna-coupled superconducting detector that can be easily incorporated with any planar antenna. Here, the attractive direction is distributed focal plane antennas [15,17]. These antennas help avoid horns or Si lenses when matching with bolometers and can be used in a multimode regime for wide-band spectrometers. In order to achieve RF matching to a distributed focal plane antenna, the series array of the CEB and JFET readouts needed to be analysed [15]. However, this configuration has several disadvantages for the spectrometer: the antenna measures only one polarisation component, the resistance is too high for matching with JFET, the Coulomb blockade starts to be important at low temperatures due to small junction capacitance and the probability of failure increases proportionally to the number of bolometers in the series array. To avoid these problems, the novel concept of the two-dimensional (2D) array of CEBs with a distributed double polarisation cross-dipole antenna is proposed. An optimal CEB with an SIN tunnel junction and Andreev contact [13] is used to overcome the aforementioned problems (Figure 1). This system is designed for the SPICA and MILLIMETRON spectrometers and other ultra-sensitive cosmology instruments.

Detection using CEB is obtained by allowing the incoming signal to pass from the antenna to the absorber through the capacitance of an SIN tunnel junction and through an SN Andreev contact. Using this optimal concept of CEB, we achieve several advantages: resistance is decreased for better matching with JFET, the Coulomb blockade is suppressed due to absence of the second junction creating SET transistor [18] and effective volume of the absorber is decreased due to proximity effect of the Andreev contact.

**Figure 2:**
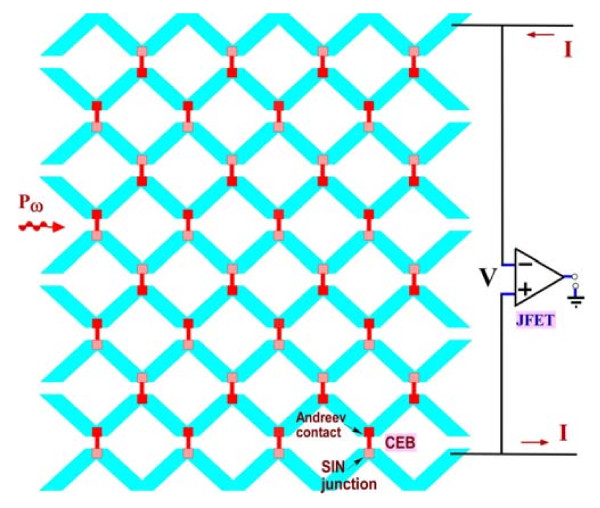
**The distributed double-polarised cross-dipole antenna with a 2D array of CEBs and a JFET readout.** This multimode double-polarised antenna is sensitive to both components of the RF signal.

The main mode of CEB operation is a concept employing a 2D array of CEBs (Figure 2) for effective matching to a JFET amplifier [16]. A distributed cross-dipole antenna (Figure 2) is proposed for receiving both polarizations of the signal in multimode operation. In this paper, we analyse a realisation of the CEB array for the 7 THz channel.

The voltage response is measured by a JFET amplifier in a current-biased mode. The main readout purpose of the 2D array is to match the total dynamic resistance of the array to the noise impedance of a JFET (approximately 0.6 MΩ). Probability of fail is considerably decreased due to parallel combination of several CEBs. For analysed array, we use four CEBs in parallel (*W* = 4) and eight rows of CEBs in series (*N* = 8).

Further performance improvements are achieved by placing four bias resistors on chips near the array of bolometers in order to decrease the noise from current-biased resistors and protect the scheme from interferences [18]. RF matching is realised by the resistance of a normal absorber, which is independent of the tunnel junction parameters.

## Methods

In our analysis of the 2D CEB array, we shall use the basic CEB model with a strong electrothermal feedback due to electron cooling [9,12,13] and the concept of series arrays in a current-biased mode [15,19]. The operation of a CEB array can be analysed using the heat balance equation for a single CEB, taking into account the power distribution between the *N* × *W* bolometers:

(1) $$\begin{array}{l} P_{\mathrm{cool}}(V,T_{e},T_{\mathrm{ph}})+\Sigma \Lambda (T_{e}^{5}-T_{\mathrm{ph}}^{5})+\frac{V^{2}}{R_{j}}+I^{2}R_{a}\\ \;=\left[P_{0}+\delta P(t)\right]/W/N \end{array}$$

Here, $\Sigma \Lambda (T_{e}^{5}-T_{\mathrm{ph}}^{5})$ is the heat flow from the electron to the phonon subsystems in the absorber, *Σ* is a material constant, *Λ* is the volume of the absorber, *T*_*e*_ and *T*_*ph*_ are, respectively, the electron and phonon temperatures of the absorber, $P_{\mathrm{cool}}(V,T_{e},T_{\mathrm{ph}})$ is the cooling power of the SIN tunnel junction, *R*_*j*_ is the subgap resistance of the tunnel junction, *R*_*a*_ is the resistance of the absorber, and *P(t)* is the incoming RF power. We can separate Eq 1 into the time independent term,

(2) $$\begin{array}{l} P_{\mathrm{SIN}0}(V,T_{e0},T_{\mathrm{ph}})+\Sigma \Lambda (T_{e0}^{5}-T_{\mathrm{ph}}^{5})\\ \;=P_{0}/W/N \end{array}$$

and the time dependent term,

(3) $$(\partial P_{\mathrm{SIN}}/\partial T+5\Sigma \Lambda T_{e}^{4})\delta T=\delta P_{1}$$

The first term in Eq. 3, $G_{\mathrm{SIN}}=\partial P_{\mathrm{SIN}}/\partial T$ , is the cooling thermal conductance of the SIN junction that gives the negative electrothermal feedback (ETF); when this term is large, it reduces the temperature response, *δT* , because the cooling power, *P*_SIN_, compensates for the change of signal power in the bolometer. The second term in Eq. 3, $G_{e-ph}=5\Sigma \Lambda T_{e}^{4}$ , is the electron–phonon thermal conductance of the absorber. From Eq. 2, we can define an effective complex thermal conductance that controls the temperature response of the CEB to the incident signal power:

(4) $$G_{\mathrm{eff}}=G_{\mathrm{SIN}}+G_{e-ph}$$

In analogy with TES [5], the effective thermal conductance of the CEB is increased by electron cooling (negative ETF). Here, we assume that the SIN tunnel junctions are current-biased, and the voltage is measured by a JFET amplifier. The responsivity, *S*_*V*_, is described by the voltage response to the incoming power:

(5) $$S_{V}=\frac{\delta V_{\omega }}{\delta P_{\omega }}=\frac{\partial V/\partial T}{G_{e-ph}+G_{\mathrm{SIN}}}$$

In the second term of Eq. 5,

(6) $$G_{\mathrm{SIN}}=\frac{\partial P_{\mathrm{SIN}}}{\partial T}-\frac{\partial P_{\mathrm{SIN}}}{\partial V}\left(\frac{\partial I}{\partial T}/\frac{\partial I}{\partial V}\right)$$

is the cooling thermal conductance of the SIN junction, which provides some electron cooling and helps to avoid the overheating of the absorber.

Noise properties are characterised by the NEP, which is the sum of three contributions:

(7) $$\begin{array}{l} NEP_{\mathrm{tot}}^{2}=N*W*NEP_{e-ph}^{2}+N*W*NEP_{\mathrm{SIN}}^{2}\\ \;+NEP_{\mathrm{AMP}}^{2}. \end{array}$$

Here, *NEP*_*e-ph*_ is the noise associated with electron–phonon interaction:

(8) $$NEP_{e-ph}^{2}=10k_{B}\Sigma \Lambda (T_{e}^{6}+T_{\mathrm{ph}}^{6})$$

In Eq. 7, NEP_SIN_ is the noise of the SIN tunnel junctions. The SIN noise has three components: the shot noise, *2eI/ S*^*2*^_*I*_, the fluctuation of the heat flow through the tunnel junctions and the correlation between these two processes:

(9) $$NEP_{\mathrm{SIN}}^{2}=\frac{\delta I_{\omega }^{2}}{{\left(\frac{\partial I}{\partial V}Sv\right)}^{2}}+2\frac{<\delta P_{\omega }\delta I_{\omega }>}{\frac{\partial I}{\partial V}Sv}+\delta P_{\omega }^{2}$$

Due to this correlation, the shot noise is increased by 30–50% in contrast to a CEB in voltage-biased mode, where strong anti-correlation decreases the shot noise.

The last term of Eq. 7 depends on the voltage *δV* and the current *δI* noise of a JFET*,* which are expressed in nV Hz^-1/2^ and pA Hz^-1/2^:

(10) $$NEP_{\mathrm{AMP}}^{2}=(\delta V^{2}+{\left(\delta I*(Rd+Ra)/W*N\right)}^{2})/{\left(S_{V}/W\right)}^{2}$$

Estimations were made for the 7 THz channel of SPICA.

## Results and discussion

**Figure 3:**
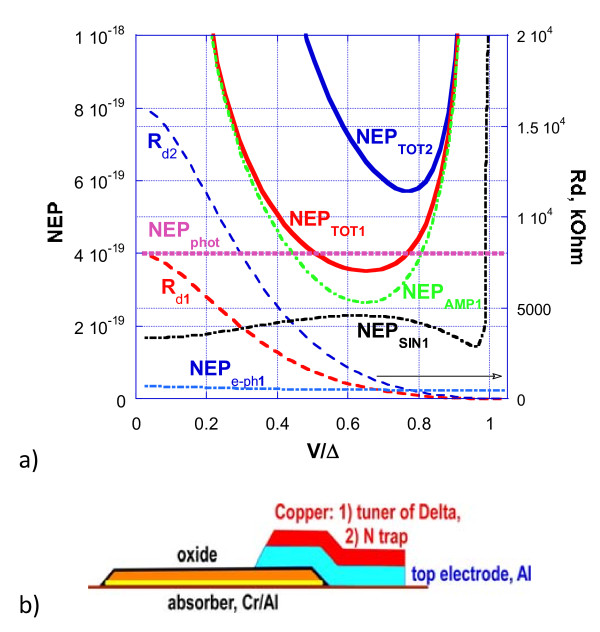
NEP components for the 2D array of CEBs with a single SIN junction and a SN Andreev contact (marked by “1”) and with a double SIN junction (marked by “2”). The array consists of four CEBs in parallel (*W* = 4) and eight CEBs in series (*N* = 8). Dynamic resistance, R_d_, is shown for both cases, on the right axis. Parameters: *f* = 7 THz, *P*_0_ = 0.02 fW, *I*_amp_ = 5 fA Hz^−1/2^, *V*_amp_ = 3 nV Hz^−1/2^ (JFET), *R* = 4 kOhm, *Λ* = 0.002 μm^3^, *T* = 70 mK, Tc = 400 mK. **b**) Fabrication of the CEB with the suppressed superconducting gap of the SIN tunnel junction.

The results of the simulation of the 2D array with JFET readout are shown in Figure 3. Figure 3 shows considerable improvement in noise properties for the CEB with a single SIN junction and an SN Andreev contact as compared to the CEB with a double SIN junction. Improvement of the NEP for a single SIN junction is achieved due to the two-fold decrease in dynamic resistance, R_d_, and a proportional decrease in the amplifier noise, as determined by the product of the amplifier current noise and the R_d_ (10). The total noise is determined mainly by the amplifier noise (JFET) and the SIN tunnel junction noise (9). The electron–phonon noise is small due to small volume of the absorber and the low temperature. As we can see from Figure 3, the noise performance for the optical power load of 0.02 fW fits the requirements of NEP_tot_ < NEP_phot_ for *R* = 4 kOhm. The simulations show that a better NEP can be obtained with a decreased gap of Al to the level of 400 mK. This suppression of the gap can be obtained by the additional evaporation of any normal metal (e.g. Cu, Ti) on the top of the counter electrode (Figure 3b).

**Figure 4:**
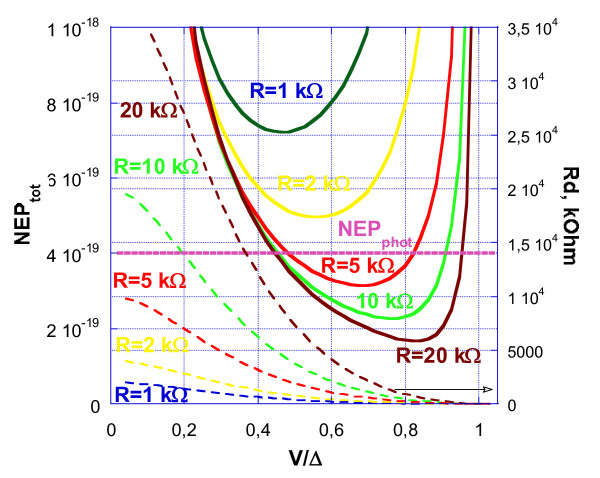
**The NEP components of the 2D array of CEBs.** NEP components of the 2D array of CEBs with JFET readout at 7 THz and a power load of 0.02 fW. Here, *I*_JFET_ = 5 fA Hz^-1/2^, *V*_JFET_ = 3 nV Hz-1/2, *Λ* = 0.002 μm^3^, *T* = 70 mK, and Tc = 400 mK for different values of normal resistance *R.*

The results of the simulation for the 2D array for different resistances of the SIN tunnel junction are shown in Figure 4. As we can see from Figure , the noise performance for the optical power load of 0.02 fW fits the requirements of SAFARI with NEP_tot_ < NEP_phot_ for *R* > 4 kOhm. The improvement of NEP for larger *R* is achieved due to the increase of responsivity *S*_*v*_ *= dV/dP*, which is proportional to *R*. For the left slope of the NEP curves in Figure 4, this improvement is compensated by an increase in amplifier noise due to the product of amplifier current noise and junction dynamic resistance. For the right slope of NEP curves, we do not have this limitation due to amplifier noise; here, the improvement is due to a slight decrease in the overheating related to the dissipation of power in a leakage resistance.

The main progress in matching with a JFET readout is achieved by the proper selection of a parallel and series combination of CEBs and a proper resistance of SIN junctions (Figure 4). Some progress in NEP is realised due to the replacement of two SIN junctions with one SIN and one SN Andreev contact (Figure 3). Better performance is also achieved due to the proper delta suppression of the top superconducting electrode to the level of 400 mK (instead of 1.2 K for clean Al) (Figure 4). Overall, the internal overheating of the CEBs by the applied voltage is decreased with arrays of any size (even with *N* > 100). It is possible that the available CMOS readout system with multiplexing from PACS or JFET multiplexer [20] can be used for matching with the CEB array.

## Conclusions

A novel concept of a 2D array of CEBs with double polarised cross-dipole antennas is proposed for spectrometer applications demanding wideband RF matching. A distributed double-polarised dipole antenna with a 2D array of CEBs inserted in the nodes of the antennas is sensitive to both components of the RF signal. The optimal concept of the CEB including a SIN tunnel junction and an SN Andreev contact is used for better performance. This concept has several advantages: it helps to realise better matching with the JFET readout, it suppresses noise related to the Coulomb blockade due to the small area of the tunnel junctions and decreases the volume of a normal absorber for further improvement in noise performance. Besides this, the reliability of a 2D array is considerably increased due to parallel and series connections of CEBs. Estimations of the CEB noise with a JFET readout have shown an opportunity to realise an NEP that is less than photon noise at 7 THz for an optical power load of 0.02 fW.

## Competing interests

The author declares no competing interests.

## Author’s contribution

The author did not provide this information.
